# The H protein of attenuated canine distemper virus is degraded via endoplasmic reticulum-associated protein degradation

**DOI:** 10.3389/fvets.2023.1214318

**Published:** 2023-07-06

**Authors:** Wenjie Wang, Zhenwei Bi, Yakun Liu, Xingxia Xia, Jing Qian, Yeping Tan, Jianjun Zhao, Suquan Song

**Affiliations:** ^1^MOE Joint International Research Laboratory of Animal Health and Food Safety, College of Veterinary Medicine, Nanjing Agricultural University, Nanjing, Jiangsu, China; ^2^Institute of Veterinary Medicine, Jiangsu Academy of Agricultural Sciences, Key Laboratory of Veterinary Biological Engineering and Technology, Ministry of Agriculture and Rural Affairs, National Center for Engineering Research of Veterinary Bio-Products, Nanjing, Jiangsu, China; ^3^College of Animal Science and Veterinary Medicine, Heilongjiang Bayi Agricultural University, Daqing, Heilongjiang, China

**Keywords:** canine distemper virus, H protein, degradation, ERAD, replication

## Abstract

Canine distemper (CD) caused by canine distemper virus (CDV) is considered a highly contagious and acutely febrile disease in various animals around the world. Endoplasmic reticulum-associated protein degradation (ERAD) is an important biological effect induced by endoplasmic reticulum (ER) stress (ERS) for the degradation of unfolded/misfolded proteins in the ER of cells. CDV H glycoprotein is translocated into the ER for post-translational modifications. The effects of CDV H and ER on each other are unclear. In this study, we found that CDV H protein induced ERS through the PERK-mediated signaling pathway. The inhibition of ERS by 4-Phenylbutyric acid (4-PBA) increased the H protein amounts of an attenuated CDV, which was reduced by dithiothreitol (DTT)-induced ERS. Further, the H protein levels were increased when ERAD was inhibited by using Eeyarestatin I or interfering E3 ligase Hrd1 in ERAD, suggesting that the attenuated CDV H protein is degraded via ERAD. ERAD involved ubiquitin-dependent proteasome degradation (UPD) and/or autophagic-lysosome degradation (ALD). The attenuated CDV H protein was ubiquitinated and significantly increased after treatment with UPD inhibitor MG132 but not ALD inhibitor chloroquine (CQ), suggesting that ERAD degrading the attenuated CDV H protein selectively depends on UPD. Moreover, the inhibition of the degradation of CDV H protein with 4-PBA or MG132 treatment increased viral replication, whereas treatment with DTT promoting degradation of H protein was found to reduce viral replication. These findings suggest that the degradation of CDV H protein via ERAD negatively affects viral replication and provide a new idea for developing CDV prevention and control strategies.

## 1. Introduction

Canine distemper virus (CDV) infection causes systemic respiratory, gastrointestinal, and neurological clinical signs, even immunosuppression, posing a serious threat to multiple species around the world (1). Canine distemper virus virion contains nucleocapsid protein (N), phosphoprotein (P), matrix protein (M), fusion protein (F), hemagglutinin protein (H), and large protein (L), which are encoded by a single-stranded negative RNA genome with a size of ~15.7 kb (2). The H and F proteins embed on the virion surface, assembling into a viral envelope. The H protein initially recognizes and adsorbs on the host receptors, then initiates the fusion processes for the virus to invade cells, and lastly, the H protein participates in the budding process of the virus (3). The H protein of CDV is the major target for neutralizing antibodies though it shows high variability (4). Thus, the CDV H protein is closely related to virus replication, pathogenicity, and immune protection. However, the degradation of CDV H protein in host cells remains poorly studied.

As a complex, dynamic organelle in cells, the endoplasmic reticulum (ER) performs a number of functions such as Ca^2+^ storage, protein folding, and lipid and carbohydrate metabolism. When proteins entering the ER cannot be effectively folded, it causes a condition known as ER stress (ERS), which plays a critical role in restoring homeostasis and normal ER function (5). Upon the accumulation of misfolded or unfolded proteins in the endoplasmic reticulum (ER) lumen, the unfolded protein response (UPR) is initiated by the activation of three major ER sensors: protein kinase R-like ER kinase (PERK), activating transcription factor 6 (ATF6), and inositol requiring enzyme 1 (IRE1) (6). UPR can promote protein folding by increasing the expression of molecular chaperones and folding enzymes, reduce protein production, and load in the ER lumen by negatively regulating the expression of secretory proteins (7). In addition, the UPR can increase the degradation of misfolded/unfolded proteins in ER, which was known as endoplasmic reticulum-associated degradation (ERAD) (8). In the ERAD process, chaperones and lectins located within the lumen of the ER recognize misfolded or unfolded proteins. These proteins are subsequently transferred to ERAD adapters which are embedded in the ER membrane, where they are ubiquitylated by ER-associated E3 ubiquitin ligases, such as Hrd1 (9). With the help of VCP/p97 and its helper proteins, ub-conjugation substrates are extracted from the ER membrane into the cytoplasm for degradation via the 26S proteasome and/or autophagy-lysosome (10, 11). During virus infection, CDV H proteins were translocated into the ER to go through post-translational modifications, such as folding, processing, and glycation (12). Therefore, it was necessary to explore the role of ERAD in the degradation of CDV H protein.

In this study, we found that CDV H protein activated the PERK signaling pathway of ERS, resulting in the ERAD of CDV H protein through the ubiquitin-dependent proteasome degradation pathway. Our findings indicated that the degradation of H protein negatively affected CDV replication, and the inhibition of H protein degradation significantly enhanced viral replication. These results explored the pivotal role of ERAD in the degradation of H protein and replication of CDV, providing a new idea for developing antiviral strategies for CDV.

## 2. Materials and methods

### 2.1. Cells, viruses, and plasmids

Vero and HEK293T cells were maintained in a humidified atmosphere containing 5% CO_2_ at 37°C and cultured in Dulbecco’s Modified Eagle’s Medium (DMEM, Gibco) supplemented with 10% fetal bovine serum (FBS) (Gibco). The CDV 851 strain was cultured in Vero cells in our laboratory with 10^5^ TCID_50_/mL (13). The plasmids pcDNA3.1-Ha-Ub and pCAGGS-Flag-H of CDV 851 strain and wild type NJ(11)2 strain were available in our laboratory (14, 15).

### 2.2. Reagents and antibodies

MG132, cycloheximide (CHX), chloroquine (CQ), 4-Phenylbutyric acid (4-PBA), and Eeyarestatin I were obtained from MedChemExpress, and Dithiothreitol (DTT) was obtained from Yeasen. The transfection reagent jetPRIME was obtained from Polyplus. The antibodies were obtained commercially: anti-Flag mouse monoclonal antibody (F1804, Sigma), anti-ATF6 rabbit polyclonal antibody (DF6009, Affinity), anti-Hrd1 rabbit polyclonal antibody (A2605, Abclonal), anti-HA mouse monoclonal antibody (BD-PM2095, Biodragon), anti-GAPDH mouse monoclonal antibody (60004-1-Ig, Proteintech), HRP-conjugated goat anti-mouse IgG (BF03001, Biodragon), and fluorescein isothiocyanate (FITC)-conjugated goat anti-mouse IgG (A0568, Beyotime). The mouse monoclonal antibody G3N against CDV NP was homemade in our laboratory (13).

### 2.3. RT-qPCR and RT-PCR

Total RNA was isolated using the RNAiso reagent (TAKARA). An equal concentration of obtained RNAs (500 ng) was reverse transcribed to cDNA through the Evo M-MLV Reverse Transcription kit (Accurate Biology) according to the manufacturer’s instructions. The synthesized cDNAs were subjected to real-time quantitative PCR using the SYBR Green Master kit (Yeasen). Specific primers (Table 1) for CHOP, GRP78, ATF4, XBP1s, XBP1u, CDV-N, and GAPDH were utilized for amplification. Quantitative PCR (qPCR) was conducted on StepOnePlus Real-Time PCR System and the relative mRNA levels were calculated by the 2^−ΔΔCT^ method. To detect XBP1 mRNA splicing, the primers XBP1u-F2 and XBP1u-R in Table 1 were used to amplify XBP1 mRNAs from synthetized cDNAs by PCR using ApexHF HS DNA Polymerase FS Master Mix (Accurate Biology) according to the manufacturer’s instruction. The examination of XBP1 mRNA splicing was performed by the restriction endonuclease *Pst* I (TAKARA) only digesting the XBP1u isoform as described in previous literature (16).

**Table 1:** Primers for (q)PCR used in this study.

Primers	Sequence (5′-3′)
GRP78-F	TCCTATGTCGCCTTCACTC
GRP78-R	ACAGACGGGTCATTCCAC
CHOP-F	CCTCCTGGAAATGAAGAGGAAG
CHOP-R	GTGACCTCTGCTGGTTCTGG
ATF4-F	CAGCAGCACCAGGCTCT
ATF4-R	TCGAAGGTGTCTTTGTCGGT
XBP1s-F	GAGTCCGCAGCAGGTGCA
XBP1s-R	CCGCCAGAATCCATGGGG
XBP1u-F1	TCCGCAGCACTCAGACTACGT
XBP1u-F2	ACGGCCTTGTAGTTGAGAACC
XBP1u-R	ATGCCCAACAGGATATCAGACTC
CDV-N-F	ACAGATGGGTGAAACAGC
CDV-N-R	CTCCAGAGCAATGGGTAG
GAPDH-F	GTGGTGCTAAGCGTGTTATCATC
GAPDH-R	GGCAGCACCTCTGCCATC

### 2.4. Immunoprecipitation

Cell transfection was conducted with jetPRIME reagent following the manufacturer’s instruction. Briefly, 293T cells were co-transfected with Flag-tagged H protein or vector and Ha-tagged Ub for 24 h. Afterward, the transfected cells were lysed in NP-40 buffer (Beyotime) for 30 min at 4°C. The lysates were then centrifuged at 12,000 × g for 10 min at 4°C, and the supernatants were collected. Subsequently, an anti-Flag mouse monoclonal antibody was added to the supernatants and incubated overnight at 4°C. Following that, protein A/G agarose (Santa Cruz) was added for 4 h incubation at 4°C. After washing, the immunoprecipitated proteins were obtained. The CDV H protein and ubiquitin in obtained samples were detected using anti-Flag and anti-Ha antibodies, respectively, by Western blot.

### 2.5. Viral titers assays

The CDV 851 strain, with a titer of 10^5^ TCID50/mL, was cultured in Vero cells in our laboratory. Viral titer assays were performed using the 50% tissue culture infectious dose (TCID_50_) method. Briefly, Vero cells were seeded in 96-well plates and incubated overnight. Serial dilutions of virus samples were added in quadruplicate to the wells and incubated with cells for 1 h at 37°C with 5% CO_2_. After the incubation, the virus inoculum was discarded, and the cells were washed three times with phosphate-buffered saline (PBS). Fresh culture medium containing 5% FBS was added to the wells, and the cells were further incubated for 3–7 days at 37°C with 5% CO_2_ until cytopathic effects (CPE) were observed in the control wells. The TCID_50_ was determined using the Reed-Muench method based on the dilution at which 50% of the wells showed CPE.

### 2.6. Cell viability assays

The cell counting kit-8 (CCK-8, Beyotime) was performed to measure cell viability following the manufacturer’s instructions. Briefly, Vero cells were seeded in 96-well plates for 24 h. Then the cells were treated with varying concentrations of MG132 (0, 2, 5, 10, 20, 40, and 80 μM) for an additional 18 h. After adding 10 μL CCK-8 reagent per well, the cell plates were shaken for 2 min and incubated for 1 h at 37°C. The absorbance was measured at a wavelength of 450 nm using an ELISA plate reader (BioTek).

### 2.7. Western blot

Cell samples were lysed in NP-40 buffer (Beyotime) for 10 min at 4°C, and the supernatants were then mixed with 5 × SDS loading buffer (Beyotime). The prepared samples were boiled for 10 min and then resolved by 10% SDS-PAGE. Subsequently, the resolved proteins were transferred to PVDF membranes. The blocking buffer (5% skim milk in PBS) was used to block the membranes at 37°C for 1 h. After washing three times with PBS containing 0.1% Tween-20 (PBST), the membranes were incubated overnight at 4°C with corresponding primary antibodies at recommended dilutions. Subsequently, the membranes were incubated with HRP-conjugated antibodies as secondary antibodies at room temperature for 1 h. After washing three times with PBST, the membranes were incubated with ECL reagent (vazyme), and the separated proteins were visualized using Tanon-5,200 Chemiluminescent Imaging System (Tanon Science & Technology Co).

### 2.8. Gene knockdown by siRNA

The siRNA targeting Hrd1 was synthesized by GenePharma (Shanghai, China) based on the following sequence: Forward: 5′-CCGCCAUGCUGCAGAUCAAtt-3′, Reverse: 5′-UUGAUCUGCAGCAUGGCG-Gtt-3′, as described in reference (17). To perform the transfection, 293T cells were seeded into 12-well plates and transfected with siHrd1 or siNC (control siRNA) using jetPRIME reagent (Polyplus) according to the manufacturer’s instructions. After 24 h, the cells were transfected with Flag-CDV H for an additional 24 h. Subsequently, Western blot analysis was performed to confirm the silence effects of Hrd1 and measure the expression of CDV H protein.

### 2.9. Immunofluorescence assays

Vero cells were infected with the CDV 851 strain in a 24-well microtiter plate and incubated for 72 h. Uninfected cells were included as a negative control. Following the incubation period, the cells were fixed with 4% paraformaldehyde at 4°C for 1 h. After blocking with PBST containing 10% FBS, the cells were incubated with monoclonal antibody G3N for 2 h at 37°C. After washing three times with PBST, the cells were incubated with fluorescein isothiocyanate (FITC)-conjugated goat anti-mouse IgG for 1 h at 37°C. Finally, the plate was washed three times with PBST and viewed by fluorescence microscope (Nikon TS100, Japan).

### 2.10. Statistical analysis

Data are expressed as the mean ± standard deviation (SD). Statistical significance was performed using GraphPad Prism 8. Band intensities were quantified using ImageJ software. The student *t*-test was employed to determine statistical differences between the two groups. In the figures, asterisks indicate statistical significance, with * representing *p* < 0.05 and ** representing *p* < 0.01.

## 3. Results

### 3.1. The H protein of CDV induced ERS

**Figure 1:**
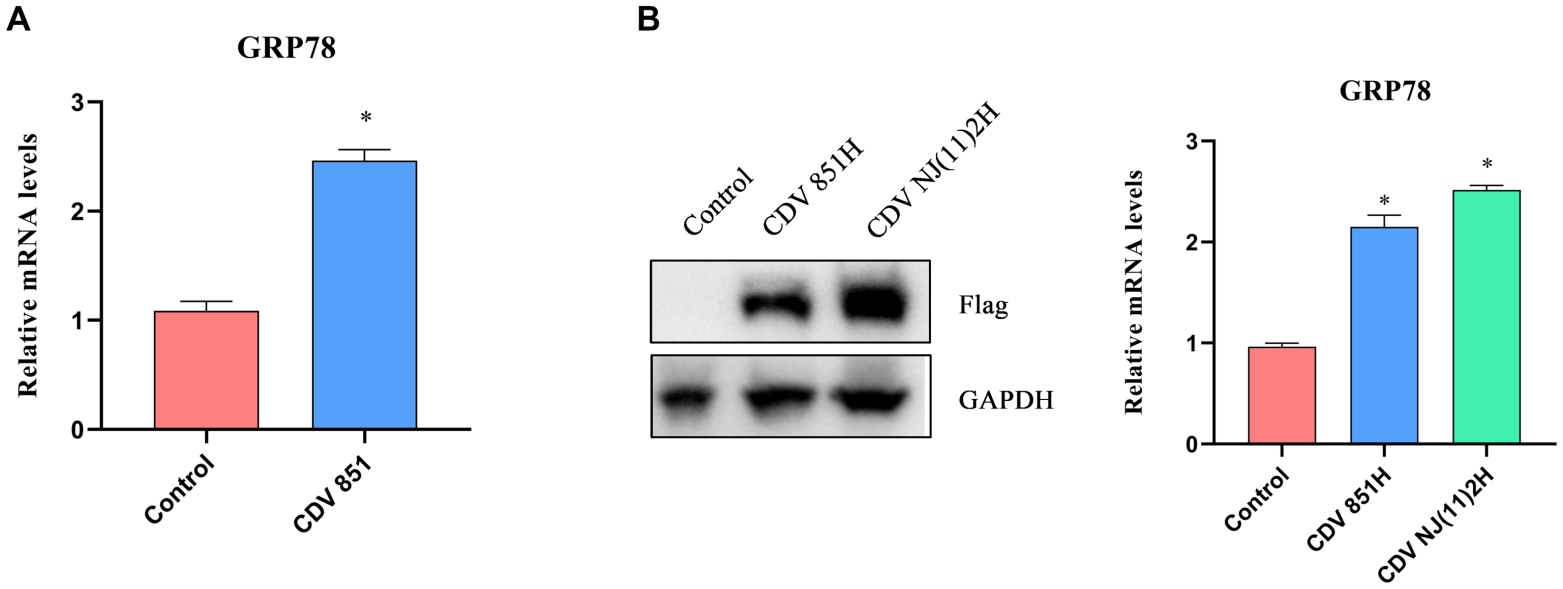
The ERS was induced by CDV H protein. **(A)** CDV infection increased the mRNA level of ERS master GRP78. Vero cells were infected with CDV 851 (MOI = 0.1) for 72 h, then the cells were harvested for RT-qPCR to examine the GRP78 mRNA level. **(B)** CDV H protein increased the mRNA level of GRP78. 293T cells were transfected with Flag-CDV H protein for 24 h; the expression of H protein was detected by Western blot, and the GRP78 mRNA level was detected by RT-qPCR. All results are presented as means ± SD obtained from at least three independent sample preparations. *p < 0.05.

It has been reported that viral replication easily induced ERS because the production of viral proteins placed an additional burden on ER (18). We examined the effect of CDV infection on ERS. Vero cells were infected with CDV 851 at a multiplicity of infection (MOI) of 0.1, and the mRNA level of GRP78, a master regulator of ERS, was assessed by RT-qPCR. The results showed that CDV infection elevated the mRNA level of GRP78 (Figure 1A), indicating that CDV infection caused ERS. CDV H protein was transported to the ER for processing and glycosylation (12). To test whether CDV H protein could induce ERS, 293T cells were transfected with Flag-CDV H protein of CDV 851 and NJ(11)2 strain for 24 h, respectively. Then the cells were harvested and the mRNA level of GRP78 was measured by RT-qPCR. The results showed that the H protein of both CDV 851 and NJ(11)2 strains increased the GRP78 mRNA level, albeit with varying degrees of intensity (Figure 1B), suggesting that CDV H protein could induce ERS. In addition, compared with CDV 851, we observed a higher H protein level of CDV NJ(11)2 (Figure 1B).

### 3.2. The signaling pathways of ERS caused by CDV H protein

**Figure 2:**
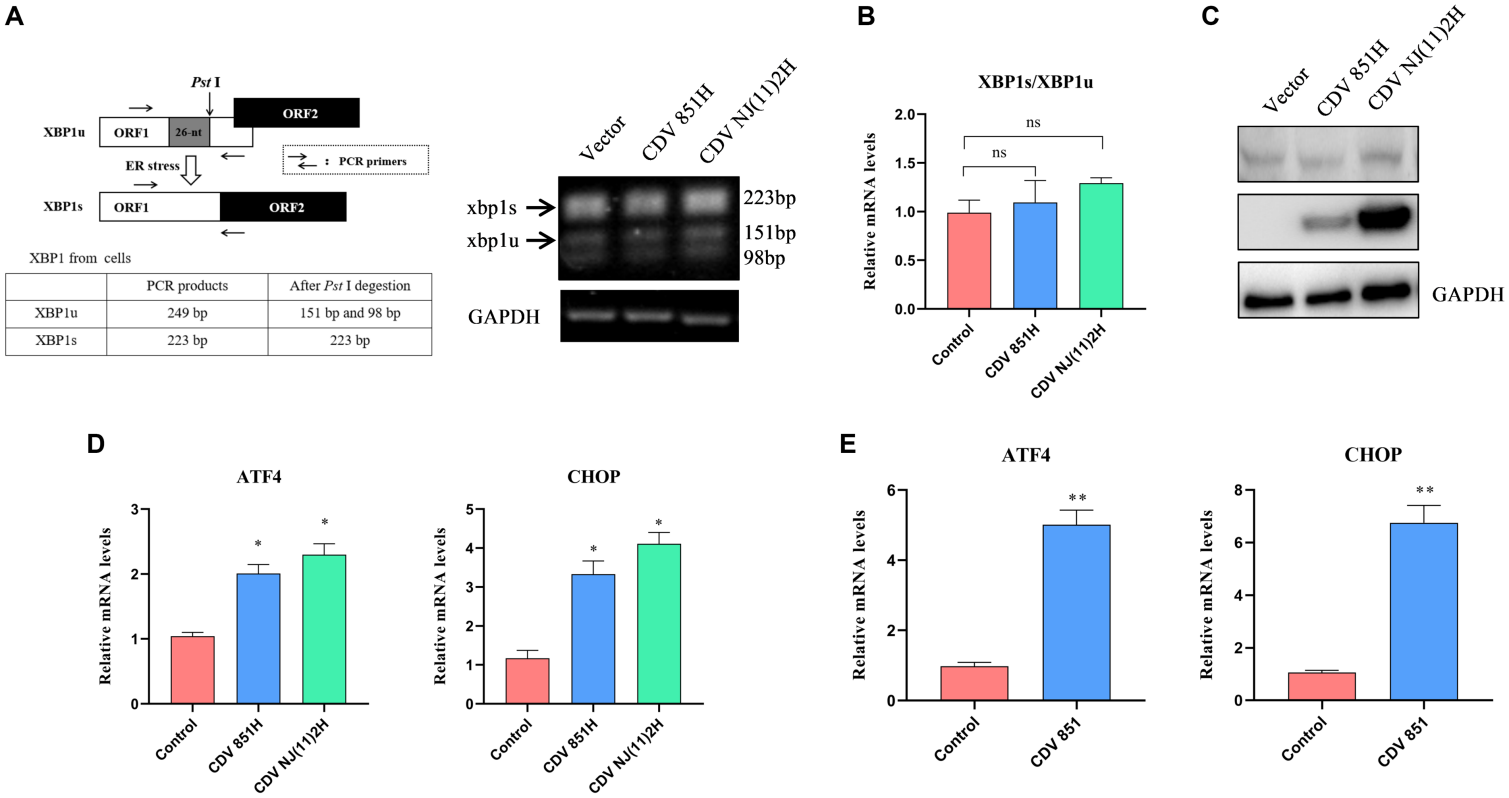
CDV H protein activated the PERK signaling pathway. **(A,B)** CDV H protein has no influence on XBP1 mRNA splicing. The analysis scheme for XBP1 mRNA splicing was shown. 293T cells were transfected with Flag-CDV H protein for 24 h; the XBP1 mRNA was amplified using RT-PCR and further digested with *Pst* I. The digested PCR products were separated by agarose gel electrophoresis **(A)**, and the relative expression level of XBP1s and XBP1u was quantified by RT-qPCR **(B)**. **(C)** CDV H protein does not affect the cleaved ATF6 (p50 ATF6). 293T cells were transfected with Flag-CDV H protein for 24 h; the expression of ATF6 protein, H protein, and GAPDH was detected by Western blot with anti-ATF6, anti-Flag, and anti-GAPDH antibodies, respectively. **(D)** CDV H protein increased the mRNA expression of the ATF4 and CHOP genes. 293T cells were transfected with Flag-CDV H protein for 24 h, then the mRNA levels of ATF4, CHOP, and GAPDH were detected by RT-qPCR. **(E)** CDV infection increased the mRNA expression of ATF4 and CHOP of the PERK pathway. Vero cells were infected with CDV 851 at MOI = 0.1 for 72 h, then were harvested, and mRNA expression of ATF4 and CHOP was quantified by RT-qPCR. All results are presented as means ± SD obtained from at least three independent sample preparations. *p < 0.05, **p < 0.01.

ERS potentially triggers PERK-, IRE1-, and ATF6-mediated signaling pathways (19). We wanted to analyze the ERS signaling pathways activated by the CDV H protein. IRE1 activation creates a spliced X-box binding protein 1 (XBP1s) by selectively cleaving a 26-nucleotide (nt) region from XBP1 mRNA by endoribonuclease activity, thus XBP1 but not XBP1s could be digested by *Pst* I, recognizing a site within the 26-nt region (20). The XBP1 products amplified from 293T cells expressing CDV H protein by RT-PCR were digested by *Pst* I, and the results suggested that the overexpression of CDV H protein has no effect on the amount of the spliced XBP1s (Figure 2A). The XBP1 mRNA was also detected by RT-qPCR, and the ratio of XBP1s/XBP1u also has no obvious change (Figure 2B). These results indicated that the CDV H protein did not induce the IRE1-mediated signaling pathway. In response to ERS, the full-length ATF6 (p90 ATF6) with 90-kDa in ER translocates to the Golgi apparatus, where it is processed to 50-kDa form (p50 ATF6) and functions as an active transcription factor (21). Our result showed that the expression of CDV H protein in 293T cells did not alter the generation of cleaved p50 ATF6 (Figure 2C), indicating that the ATF6-mediated pathway was not activated by CDV H protein. In the PERK signaling pathway, activated PERK phosphorylates eIF2α, which increases the transcription level of the downstream molecular CHOP and ATF4 of eIF2α (22). Thus, we determined the transcription level of CHOP and ATF4 in 293T cells expressing CDV H protein by RT-qPCR. As shown in Figure 2D, the expression of CDV H protein resulted in an elevated mRNA level of the ATF4 and CHOP, suggesting that CDV H protein activated the PERK-eIF2α-ATF4-CHOP pathway. Similar to the H protein, CDV infection also elevated the mRNA levels of ATF4 and CHOP (Figure 2E).

### 3.3. The effect of ERS on CDV H protein

**Figure 3:**
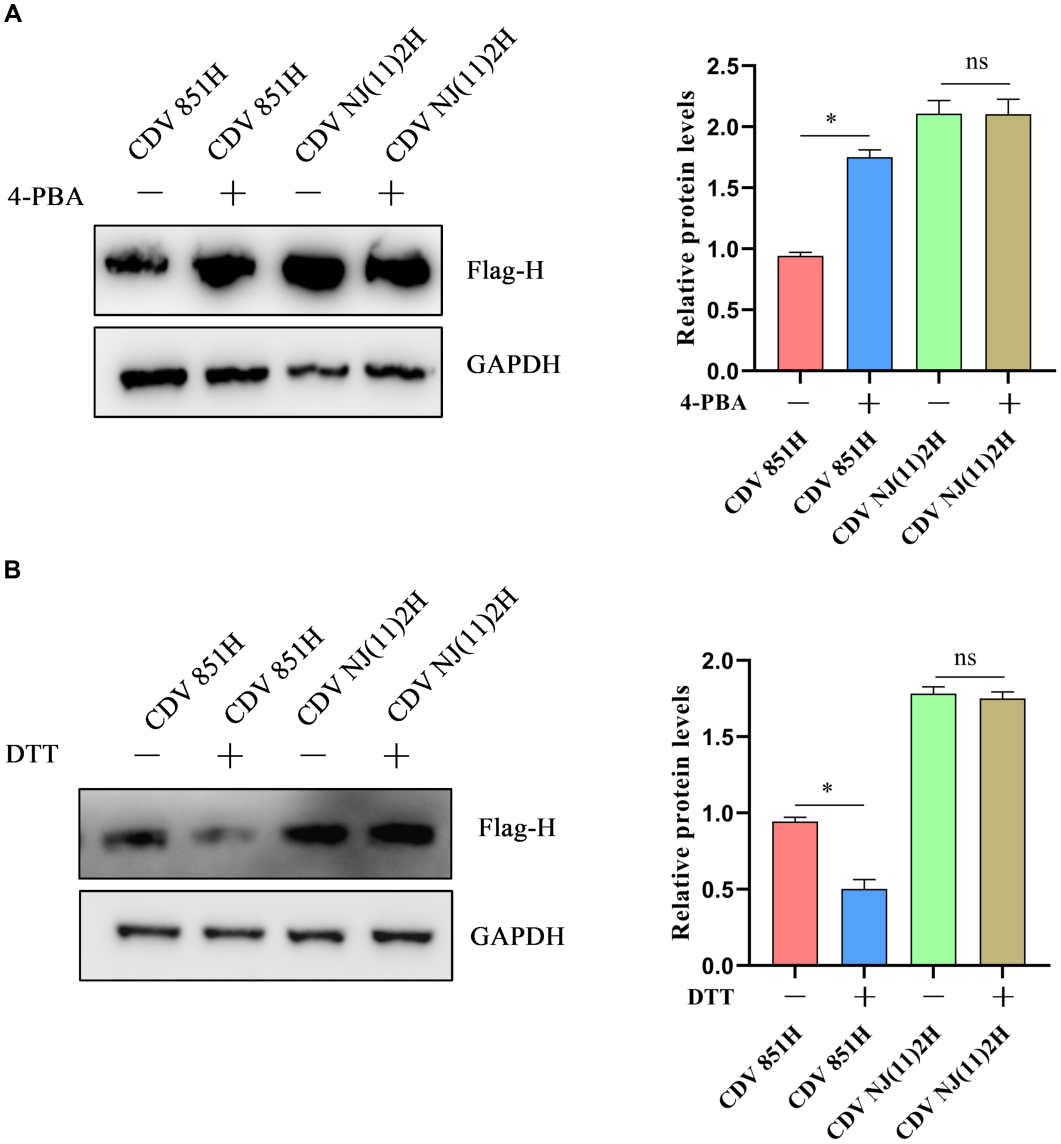
The effect of ERS on CDV H protein. **(A)** Inhibition of ERS promoted the H protein level of CDV 851 but not CDV NJ(11)2. 293T cells were transfected with Flag-CDV H for 24 h, then were treated with 4-PBA (0.5 mM) for 4 h. **(B)** The induction of ERS decreased the H protein level of CDV 851 but not CDV NJ(11)2. 293T cells were transfected with Flag-CDV H for 24 h, then were treated with DTT (1 mM) for 1 h. The cell lysates were analyzed by Western blot using anti-Flag and anti-GAPDH antibodies. The gray values of protein bands were analyzed by ImageJ software and the ratio of target protein gray values to GAPDH was calculated. All results are presented as means ± SD obtained from at least three independent sample preparations. *p < 0.05.

ERS signaling cascades are activated to counteract the impairment by resolving protein folding defects, attenuating protein synthesis, and degrading unfolded/misfolded proteins (23). To identify the impact of ERS on CDV H protein level, 293T cells were transfected with Flag-CDV H protein for 24 h, and the H protein levels were assessed after treatment with the ERS inhibitor 4-PBA for 4 h. The results of the Western blot showed the H protein level of CDV 851 was increased while CDV NJ(11)2 was unchanged (Figure 3A). Furthermore, 293T cells were transfected with Flag-CDV H protein for 24 h and the H protein level was assessed after treatment with the ERS inductor DTT for 1 h. The results showed that the H protein level of CDV 851 was decreased while CDV NJ(11)2 remained unchanged (Figure 3B). These results suggested that ERS reduced the H protein levels of the attenuated CDV strain.

### 3.4. The H protein of attenuated CDV was degraded via ERAD

**Figure 4:**
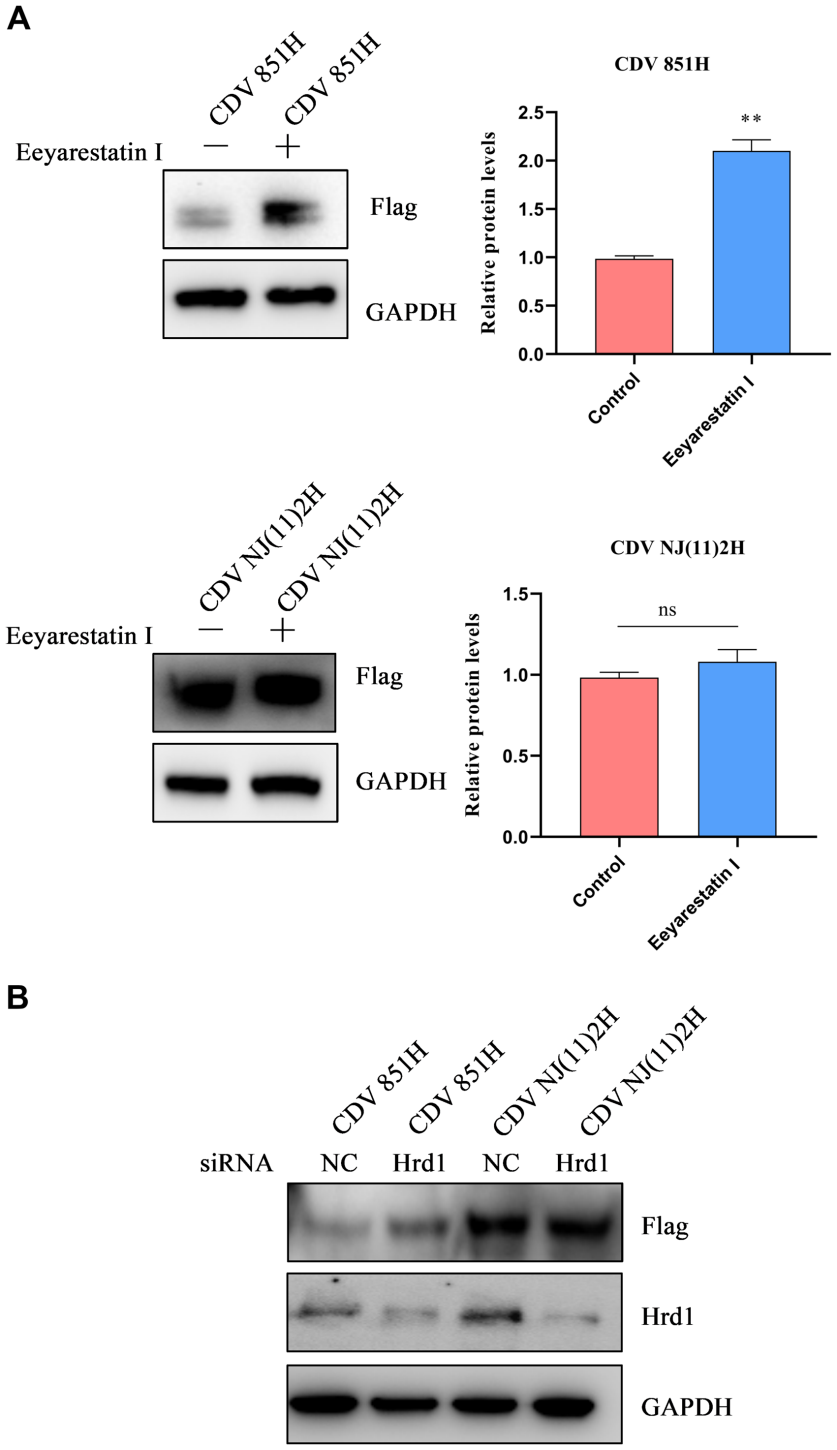
The degradation of CDV 851H protein via ERAD. **(A)** The inhibition of ERAD by Eevarestatin I increased the CDV 851H protein level. 293T cells were transfected with Flag-CDV H for 24 h, then were treated with Eevarestatin I (4 μM) for 4 h. The cell lysates were analyzed by Western blot using anti-Flag and anti-GAPDH antibodies. The gray values of protein bands were analyzed by ImageJ software and the ratio of target protein gray values to GAPDH was calculated. **(B)** Knockdown of Hrd1 increased the CDV 851H protein level. After transfecting 293T cells with siRNA for Hrd1 or si-NC for 24 h, the cells were transfected with H protein of CDV 851 and CDV NJ(11)2 for another 24 h. Western blot was used to detect the CDV H protein level and confirm the silencing efficacy of Hrd1-targeted siRNA. The gray values of protein bands were analyzed by ImageJ software and the ratio of target protein gray values to GAPDH was calculated. All results are presented as means ± SD obtained from at least three independent sample preparations. *p < 0.05, **p < 0.01.

Under ERS, unfolded/misfolded proteins are often degraded by the ERAD to maintain homeostasis (24). To identify whether CDV H protein was degraded via ERAD, an ERAD inhibitor Eevarestatin I was selected to treat 293T cells transfected with Flag-CDV H protein. The results showed the H protein level of CDV 851 was apparently elevated, while CDV NJ(11)2H protein level remained unchanged (Figure 4A). Furthermore, an RNA interference (RNAi) approach was utilized to silence Hrd1, a key E3 ligase for ERAD. 293T cells were transfected with siRNA for Hrd1 or si-NC for 24 h. Subsequently, the cells were further transfected with the H protein of CDV 851 and CDV NJ(11)2 for an additional 24 h. Western blot was used to detect the CDV H protein level and confirm the silencing efficacy of Hrd1-targeted siRNA. As shown in Figure 4B, the depletion of Hrd1 protein resulted in the restoration of CDV851 H protein levels, while no effect was observed on CDV NJ(11)2H. It further suggested that CDV 851H protein was degraded through ERAD. Collectively, these results demonstrated that CDV 851H protein was degraded through ERAD, whereas CDV NJ(11)2 antagonized the degradation.

### 3.5. Degradation of attenuated CDV H protein depended on UPD

**Figure 5:**
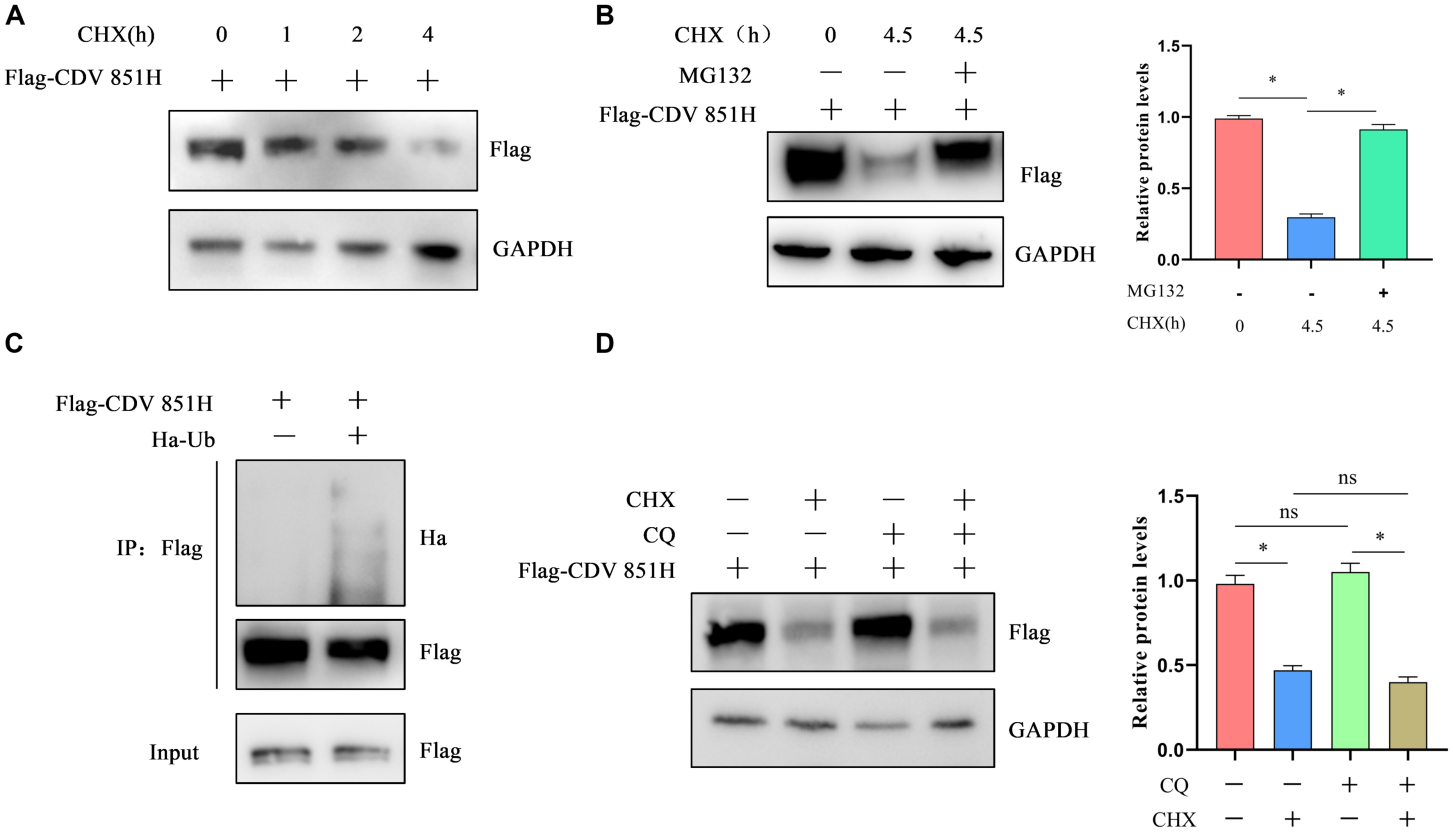
The degradation of CDV 851H protein depended on the ubiquitin-proteasome pathway. **(A)** CDV 851H protein was degraded in cells. 293T cells were transfected with Flag-CDV H for 24 h, then were treated with CHX (50 μg/mL) and collected at the indicated time points. The cell lysates were analyzed by Western blot using anti-Flag and anti-GAPDH antibodies. **(B)** CDV 851H protein was degraded by the proteasome. 293T cells were transfected with Flag-CDV 851H without or with MG132 (20 μM) and CHX (50 μg/mL) for 4.5 h and collected at the indicated time points. Cell lysates were analyzed by Western blot using anti-Flag and anti-GAPDH antibodies. The gray values of protein bands were analyzed by ImageJ software and the ratio of target protein gray values to GAPDH was calculated. **(C)** CDV 851H protein was ubiquitinated. 293T cells were transfected with Ha-ub and Flag-CDV 851H protein, then the transfected cells were lysed, and immunoprecipitation was performed using an anti-Flag antibody. The input samples and co-precipitated proteins were analyzed by Western blot using anti-Flag and anti-Ha antibodies. **(D)** CDV 851H protein was not degraded by the autophagic lysosomal pathway. 293T cells were transfected with Flag-CDV 851H for 24 h, then were treated with CHX (50 μg/mL) or CQ (10 mM) for 4 h; the change in CDV 851H protein was examined by Western blot using anti-Flag and anti GAPDH antibodies. The gray values of protein bands were analyzed by ImageJ software and the ratio of target protein gray values to GAPDH is shown. All results are presented as means ± SD obtained from at least three independent sample preparations. *p < 0.05.

The CDV 851H protein has been identified to be degraded via ERAD. Two major pathways are responsible for ERAD: autophagy-lysosome degradation (ALD) and ubiquitin-dependent 26S proteasome degradation (UPD) pathways (25). We further investigated the potential roles of the UPD and ALD in the degradation of CDV H protein. 293T cells were transfected with Flag-CDV 851H protein for 24 h, and the H protein level was assessed at 1, 2, and 4 h after treatment with CHX, a protein biosynthesis inhibitor. The results of Western blot showed that the CDV 851H protein level was gradually decreased in CHX-treated cells (Figure 5A). These results confirmed that CDV 851 H protein was degraded in cells. 293T cells were transfected with Flag-CDV 851H protein for 24 h, then the H protein in CHX-treated cells was examined in the presence or absence of proteasome inhibitor MG132 by Western blot. The results showed that the reduction of CDV 851H protein was significantly impaired by the treatment of MG132 (Figure 5B), suggesting that this reduction was caused by proteasomal degradation. In addition, we found that CDV 851H protein underwent ubiquitination by co-transfecting CDV 851H and ubiquitin plasmid in an IP assay (Figure 5C). Also, we tested whether CDV 851H protein could be degraded by the lysosomal pathway. 293T cells were transfected with Flag-CDV 851H protein for 24 h, then were treated with CHX and lysosome inhibitor CQ for 4 h; the expression of H protein of CDV 851 was detected by Western blot. The results indicated that the H protein level of CDV851 was not obviously different in the presence or absence of CQ (Figure 5D). It rules out the degradation of CDV 851H protein via the ALD. Collectively, the degradation of CDV 851H protein via ERAD depends on UPD but not ALD.

### 3.6. ERS regulated H protein of CDV to affect viral replication

**Figure 6:**
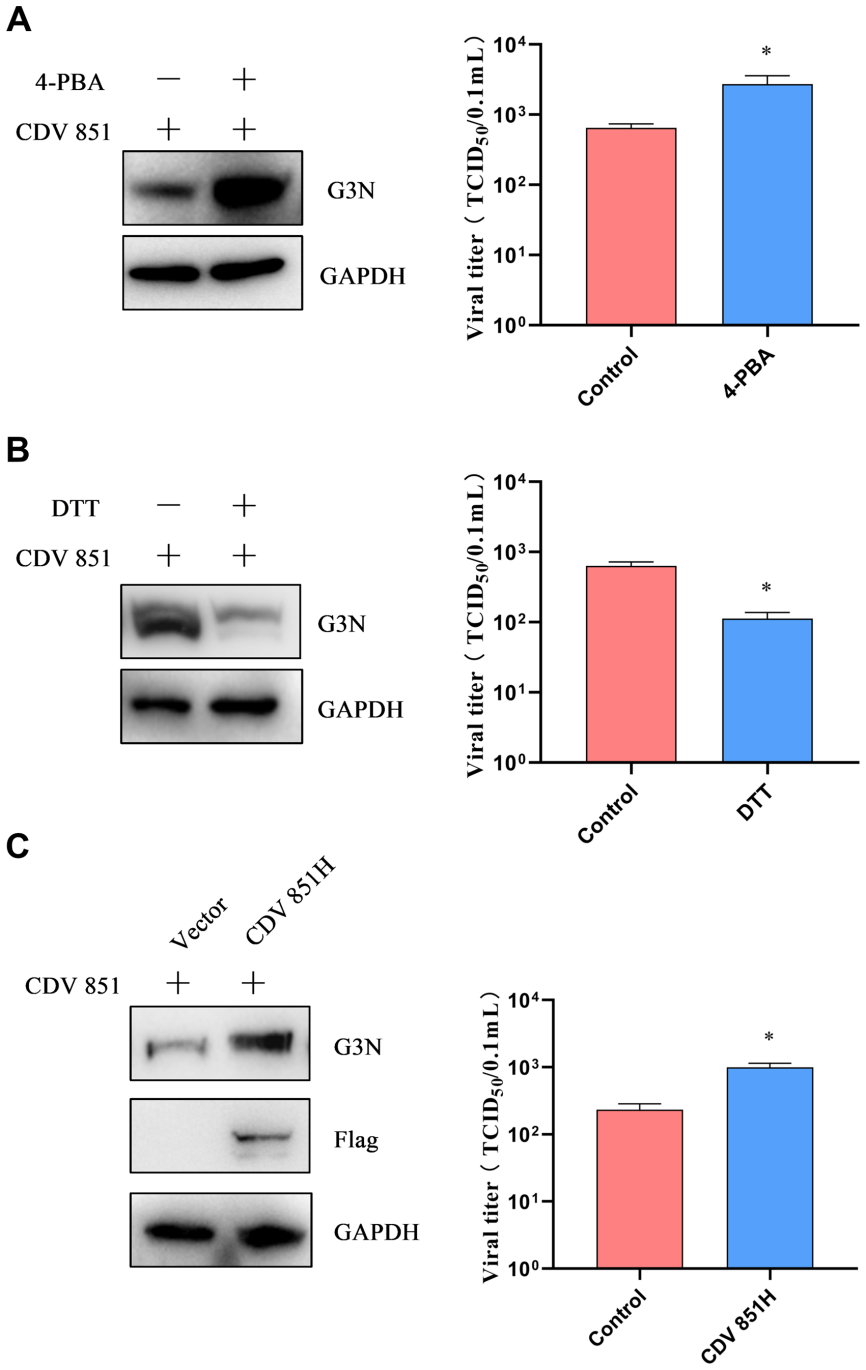
ERS regulated H protein of CDV to affect viral replication **(A,B)**. The inhibition or induction of ERS affected viral replication. Vero cells were infected with CDV 851 (MOI = 0.1) and treated with 4-PBA (0.5 mM) **(A)** or DTT (1 mM) **(B)** for 72 h, then the cells and supernatants were collected. The CDV N protein levels were detected by Western blot, and the viral titers of culture supernatants were detected by the Reed–Muench method. **(C)** Overexpression of CDV 851H increased viral replication. Vero cells were transfected with Flag-CDV 851H for 6 h, followed by infection with CDV 851 (MOI = 0.1) for 72 h. The infected cells were then analyzed by Western blot using anti-CDV N, anti-Flag, and anti-GAPDH antibodies. The viral titers in the culture supernatants were determined using the Reed-Muench method. All results are presented as means ± SD obtained from at least three independent sample preparations. *p < 0.05.

After inducing ERS, CDV 851H protein was degraded via ERAD. We sought to investigate the effects of the H protein regulated by ERS on viral replication. Vero cells were infected with CDV 851 (MOI = 0.1) and treated with 4-PBA or DTT for 72 h. The results showed that the 4-PBA treatment enhanced viral N protein levels and viral titers (Figure 6A). By contrast, the DTT treatment decreased viral N protein levels and viral titers (Figure 6B). To further confirm the contribution of H protein to viral production, Vero cells were transfected with Flag-CDV 851H for 6 h, and then infected with CDV 851 (MOI = 0.1) for 72 h. The results showed that the overexpression of CDV 851H promoted viral replication, which was evidenced by the increased N protein levels and viral titers (Figure 6C). These results demonstrated that ERS played a negative regulatory role in viral replication by one way of reducing the H protein level.

### 3.7. The block of CDV H protein degradation affected viral replication

**Figure 7:**
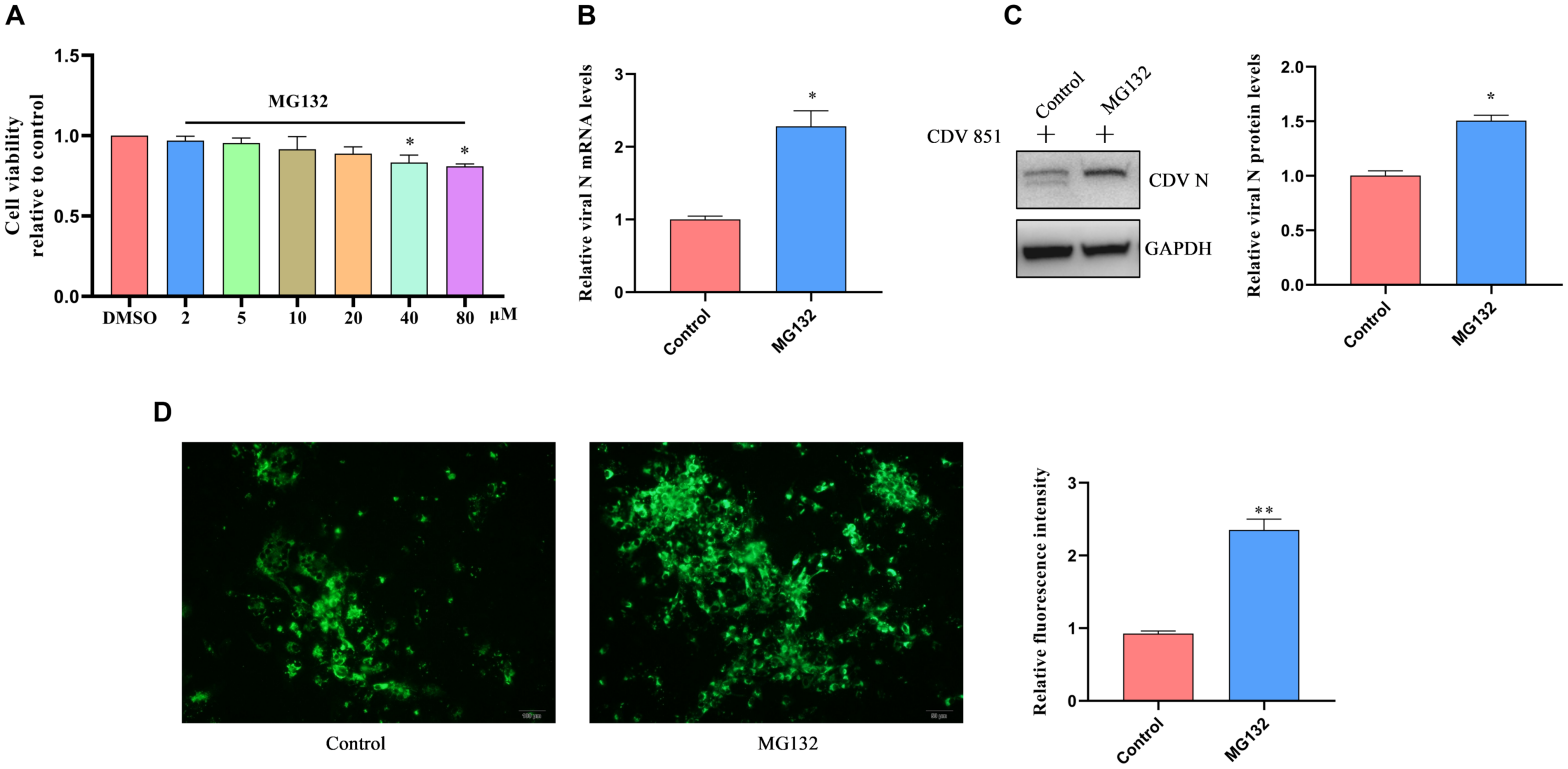
The block of CDV H protein degradation affected viral replication. **(A)** Cell viability assay of Vero cells treated with MG132 at different concentrations. Vero cells were treated with varying concentrations of MG132 for 18 h. Cell viability after treatment was evaluated using the CCK-8 assay. The absorbance values of the Vero cells treated with DMSO were used as the reference and set as 1. **(B)** Proteasome inhibitor treatment increased viral mRNA level. Vero cells were infected with CDV 851 (MOI = 0.1) for 54 h and treated with MG132 for an additional 18 h. The RT-qPCR was used for the examination of CDV N mRNA level. **(C)** Proteasome inhibitor treatment promoted viral protein levels. Vero cells were infected with CDV 851 at MOI = 0.1, cells were harvested at 72 h after treatment with MG132 for 18 h. The lysates of harvested cells were subjected to Western blot analysis using anti-CDV N and anti-GAPDH antibodies. The gray values of protein bands were analyzed by ImageJ software and the ratio of target protein gray values to GAPDH is shown. **(D)** Proteasome inhibitor treatment increased viral replication. Vero cells were infected with CDV 851 at MOI = 0.1; cells were fixed at 72 h after treatment with MG132 for 18 h. CDV N protein was detected by monoclonal antibody G3N in IFA. The green fluorescence values of CDV N protein were analyzed by ImageJ software. All results are presented as means ± SD obtained from at least three independent sample preparations. *p < 0.05, **p < 0.01.

Given that the degradation of H protein via ERAD depends on UPD, we aimed to clarify the effect of H protein degradation on CDV replication by blocking the UPD pathway using MG132. First, Vero cells were incubated with varying concentrations of MG132 for 18 h; the viability of cells was detected by the CCK-8 kit. We determined that the highest concentration of MG132 that did not affect cell viability was 20 μM (Figure 7A). Subsequently, Vero cells were infected with CDV 851 (MOI = 0.1) for 60 h, then were treated with MG132 (20 μM) for 18 h; the mRNA level of CDV N was detected by RT-qPCR. The results showed that the CDV N mRNA level was increased in the MG132-treated group compared to DMSO-treated cells (Figure 7B). The effects of MG132 on viral protein expression were also detected by Western blot. Compared with the DMSO-treated group, the expression amount of N protein was increased in the MG132-treated group (Figure 7C). Finally, the viral loads were identified by IFA, which also showed that MG132 treatment could enhance virus propagation (Figure 7D). These findings suggested that the inhibition of the degradation of attenuated CDV H protein has a positive role in viral replication.

## 4. Discussion

Many soluble and membrane proteins are located in the ER of cells for synthesis, folding, modification, and transport (26, 27); unfolded/misfolded proteins can cause ERS, which impairs ER homeostasis (7, 28). Research has found that viral replication easily induced ERS because the production of viral proteins placed a burden on the ER (18). In this study, we first found that CDV infection caused ERS. Previous studies have reported that the CDV H protein undergoes transfer into the ER to modulate protein maturation and surface transport (12). Further, we first found that the expression of the H protein elicited ERS. Our findings suggest that CDV H protein is not completely and properly folded in the ER of cells, which is detected by host cells as unfolded/misfolded or “nonself” protein. Upon ERS, three primary sensors PERK, ATF6, and IRE1 are activated, subsequently triggering a series of signaling cascades known as the UPR (29). We found that CDV H protein only activated the PERK-eIF2α-ATF4-CHOP pathway.

ERS signaling cascades are activated to counteract the impairment of ER homeostasis by resolving protein folding defects, attenuating protein synthesis, and degrading unfolded/misfolded proteins (23). The unfolded/misfolded proteins synthesized at the ER are recognized as target substrates for the ERAD pathway (30). ERAD is an important biological effect induced by ERS for the degradation of unfolded/misfolded proteins in the ER of cells (31). During influenza A viruses (IAV) infection, HA glycoprotein induced ERS, resulting in HA degradation via ERAD and consequent inhibition of IAV replication (32). After causing ERS, the NS protein of Japanese encephalitis virus (JEV) and dengue virus (DENV) were rapidly and selectively degraded by the ERAD pathway (17). The glycoprotein O (gO), a viral envelope glycoprotein of human cytomegalovirus (HCMV) could be degraded by ERAD (33). The hepatitis B virus (HBV) and hepatitis C virus (HCV) can activate ERAD to degrade viral glycoproteins, thereby reducing the viral particle and maintaining a chronic infection status (34, 35). In this study, CDV 851 H protein was identified to be degraded via ERAD. The ERAD degradation of unfolded/misfolded proteins was carried out via ubiquitin-dependent 26S proteasome degradation (UPD) and/or autophagic-lysosome degradation (ALD) (25). In this study, we found that CDV 851 H protein was modified by ubiquitin and degraded by UPD rather than ALD. As the requisite of viral replication, the CDV H protein is involved in receptor binding, mediating membrane fusion, virus budding, and virus assembly (36). This supported our observation that the degradation of the H protein impaired the replication of the virus. It has been reported that CDV H protein has various effects on cell biological functions, such as oxidative stress, apoptosis, and so on (37–39). The degradation of the H protein may contribute to viral replication and pathogenesis of CDV by regulating the biological functions of the infected cells.

CDV 851 was an attenuated strain while the NJ(11)2 strain was isolated from infected and clinically sick dogs; their genetic variation rate of H genes exhibits up to 8.7% (15). Research has reported that the amino acid sequence variation in the spike proteins of different transmissible gastroenteritis virus (TGEV) strains led to the activation of the different UPR pathways (40). We found that both strains activated the same signaling pathways, albeit with varying degrees of intensity. The CDV NJ(11)2 strain H protein level was also observed to be higher than the CDV strain (Figure 1B). In contrast to the CDV 851 attenuated strain, the CDV NJ(11)2 virulent strain exhibits resistance to ERAD degradation; the wild type CDV NJ(11)2 strain having a stronger stimulating effect on ERS may be due to more H protein level of the wild type. The activation of multiple UPR pathways has been reported in various viral infections, which are involved in viral replication and pathogenesis. All three UPR signaling pathways were activated in TGEV infection, while only activation of the PERK-eIF2α pathway largely suppressed the TEGV replication (40). Infection of IAV induced ERS and activated IRE1/XBP1 signaling pathway to degrade SOD and Sp1 through the ERAD pathway (41). Differences in ERS levels may be associated with different virulence and pathogenesis of CDV strains, and a clear understanding of the mechanism needs to be elucidated in future studies.

In a word, folding and modifications of CDV H glycoprotein in the ER caused ERS, resulting in the degradation of H protein by ERAD and the inhibition of viral replication. Our work reveals the critical role of the ERAD in CDV replication and provides a novel idea for developing new CDV prevention and control strategies.

## Data availability statement

The original contributions presented in the study are included in the article/supplementary material, further inquiries can be directed to the corresponding authors.

## Author contributions

WW, ZB, and SS contributed to the study’s conception and design. WW performed the experiments and drafted the manuscript. SS, YL, XX, JZ, JQ, and YT helped to analyze the data. ZB and SS supervised the study and designed the experiments. All authors read and approved the final manuscript.

## Funding

This work was supported by the National Natural Science Foundation of China (31802168), the National Natural Science Foundation of China (31972714), and the Open Project Program of Jiangsu Key Laboratory of Zoonosis (No. R2101).

## Conflict of interest

The authors declare that the research was conducted in the absence of any commercial or financial relationships that could be construed as a potential conflict of interest.

## Publisher’s note

All claims expressed in this article are solely those of the authors and do not necessarily represent those of their affiliated organizations, or those of the publisher, the editors and the reviewers. Any product that may be evaluated in this article, or claim that may be made by its manufacturer, is not guaranteed or endorsed by the publisher.
